# Steering the digital transformation of service-oriented organizations by job crafting and innovative work behavior: employee-driven adaptability

**DOI:** 10.3389/fpsyg.2026.1764449

**Published:** 2026-05-08

**Authors:** Qiuxian Ye, Fangli Liu, Yanqing Yan, Shuxian Ye

**Affiliations:** 1 Guangdong Fengwo Wire Trough Co., Ltd, Foshan, China; 2 Zhuhai College of Science and Technology, Zhuhai, China; 3 Department of Education and Psychological Sciences, Yuncheng University, Yuncheng, China; 4 Sports Department, Guangdong University of Science and Technology, Dongguan, China

**Keywords:** digital leadership, digital transformation, digital-enabled task performance, innovative work behavior, job crafting

## Abstract

In the digital economy, digital leadership has become a key driver of organizational change and performance improvement. However, prior research has paid limited attention to employees’ proactive behaviors, such as job crafting and innovative work behavior in the digital transformation. Drawing on dynamic capability theory and the resource-based view, this study investigated the impact of digital leadership on task performance through two mechanisms. Based on the questionnaire survey data of 415 hotel employees in China’s service industry, the study found that job crafting and innovative work behavior played significant mediating roles between digital leadership and digital transformation, which enhanced digital-enabled task performance. The research conclusion reveals the behavioral pathways through which leadership supports digital transformation and offers practical implications for enterprises to cultivate active employees, enhance digital adaptability and improve performance.

## 1 Introduction

Digitalization has reshaped business environments worldwide. Sustained improvements in employee performance are widely regarded as a key source for organizations to maintain long-term competitive advantage and achieve sustainable development (Chatterjee et al., 2023; Qiao et al., 2024). Meanwhile, technological change has become increasingly complex. In order to maintain strategic agility, ensure efficient operation and future growth, it is almost an inevitable choice for enterprises to promote digital transformation (Xue et al., 2025). However, digital transformation provides huge opportunities while also intensifying organizational challenges. Specifically, the gap between employees’ actual performance and organizational expectations continues to widen, weakening the overall effectiveness of change efforts. In order to bridge this gap, organizations must adopt a proactive leadership style, mobilize employees’ initiative and unlock their potential (Odai et al., 2026). In this context, digital leadership is often regarded as a key driver of change. Through a clear digital vision and the provision of corresponding resources, digital leadership can encourage employees to actively reshape their work and engage in innovative behavior, so as to create value and enhance organizational adaptability (Wang et al., 2025b; Zhu et al., 2022; Zia et al., 2025). In the service industry, especially in the hospitality industry, digitalization has moved far beyond a supporting role. On the contrary, it has become a strategic lever to improve service quality, business flexibility and customer participation (Busulwa et al., 2022; He et al., 2023). Although the investment in the digital transformation of the service industry is increasing, the employee-level mechanisms remain underexplored. Specifically, it is not clear how digital leaders can motivate employees through capacity-building and role adaptation to promote organizational transformation and improve performance. Recent studies, such as Wang et al. (2025a), emphasize how leadership affects employee engagement and performance, and provide valuable insights into the role of digital leadership in promoting the transformation of the hospitality industry.

In service industries, developing high-performing employees depends heavily on leadership, especially the ability of leaders to stimulate employees’ proactive behavior and guide strategic transformation (Qiao et al., 2024; Zia et al., 2025). This capability is central to digital leadership. Existing research shows that digital leadership not only helps organizations maintain growth and operational performance in a volatile environment, but also enables companies to seize strategic opportunities in the highly competitive service market (Probojakti et al., 2025). Recent research further shows that the demand for digital leadership in the hospitality industry continues to rise, highlighting the need for more in-depth academic exploration of this leadership style in the service environment (Borah et al., 2022). Therefore, bringing digital leadership into the theoretical framework helps to fill the gaps in previous research and deepen understanding of digital transformation in hotel organizations.

Digital leadership is an important driver of digital transformation. Based on the Dynamic Capability Theory (DCT; Teece et al., 1997) and Resource-Based View (RBV; Barney, 1991), this study takes employees’ job crafting and innovative work behavior as key mechanisms linking leadership to organizational transformation and adaptation. When the organization provides sufficient technical tools and support, employees are more likely to actively reorganize tasks, try new methods, and use digital resources to improve their work (Wang et al., 2025b). This proactive behavior also helps to attract external stakeholders and cultivate a positive atmosphere for organizational transformation (Zia et al., 2025). Although previous studies have independently verified that job crafting and innovative work behaviors help organizations embrace transformation (Ahmed et al., 2024; Petrou et al., 2012), systematic empirical evidence explaining how these behaviors work cooperatively between digital leadership and transformation outcomes is still limited. To fill this gap, this research constructed a parallel mediation model to test how employee behavior mediates the relationship between digital leadership and digital transformation, as well as their expanded impact on digital-enabled task performance.

In addition, the existing research on the behavior mechanism affecting employee performance is relatively scattered (Sagbas et al., 2023). Therefore, a comprehensive theoretical framework integrating employee behavior, digital transformation and task performance needs to be fully developed. This fragmentation limits our understanding of how different proactive employee behaviors interact and ultimately shape performance outcomes. Accordingly, this study explores how digital leadership can fosters employees’ job crafting and innovative work behavior, thereby supporting digital transformation of hotel enterprises and improve employees’ task performance.

In order to fill these research gaps and provide a comprehensive perspective for performance improvement in the digital age, this study focuses on leadership practices that activate employee initiative. Specifically, it addresses the following research questions:

RQ1: How does digital leadership affect digital transformation?

RQ2: How does digital leadership interact with digital transformation, to what extent do job crafting and innovative work behavior mediate this relationship?

RQ3: How does digital transformation improve employee task performance?

In theory, this study is based on DCT and RBV (Barney, 1991; Teece et al., 1997) contributes to the literature through theoretical development and empirical testing. By making a clear distinction between digital leadership driven by strategy and behavior and digital transformation as an organizational process, this study avoids conceptual overlap and clarifies their respective theoretical roles. Specifically, drawing on DCT, we conceptualize employee job crafting and innovative work behavior as dynamic capabilities at the micro level, enabling organizations to perceive, grasp., and reconfigure resources to cope with digital changes. From the perspective of RBV, these employee behaviors are further theoreticalized as valuable and difficult-to-imitate human and behavioral resources, which help to obtain sustainable performance advantages. Importantly, this study introduces a parallel chain mediation mechanism to explain how digital leadership translates into digital transformation and task performance through distinct yet complementary employee behavior paths. By integrating DCT and RBV theories (Barney, 1991; Teece et al., 1997) at the employee level, this study explores how leadership-driven digital initiatives can be translated into organizational outcomes and provides a more precise and theoretical-driven explanation. In this process, this study extends the leadership theory to the context of digital services, and provides a clearer theoretical positioning for understanding digital transformation as a behavior-based and capability-based process.

## 2 Theoretical lens and hypothesis development

### 2.1 Digital leadership and digital transformation

Digital leadership is a leadership style characterized by the active adoption of digital technologies, vision-oriented strategies, and the ability to coordinate technological change, thereby enhancing organizational agility, innovation, and performance (AlNuaimi et al., 2022; Benitez et al., 2022; Khan et al., 2024). This leadership style allows companies to combine digital vision with internal capabilities and resources (such as technological infrastructure) and that is crucial for navigating the complex process of digital transformation (Dióssy et al., 2025; Schiuma et al., 2022). Digital leadership strategically reconstructs existing organizational practices into digitally driven processes, laying the foundation for future-oriented transformation and continuous adaptability (AlNuaimi et al., 2022; Held et al., 2025). The research points out that in the context of rapid technological change, digital leadership helps organizations seize digital opportunities and promote innovation by promoting knowledge sharing and capacity reconstruction (Fatima and Masood, 2024). According to DCT (Teece et al., 1997) and RBV (Barney, 1991), digital leadership plays an important role in using adaptability and strategic resources to promote digital transformation. DCT emphasizes how organizations can constantly adjust and reallocate their resources through leadership to cope with the dynamic environment and promote organizational learning and change. This adaptive capability is critical to implementing and sustaining digital transformation because it enables organizations to restructure their systems, processes, and service models (Chatterjee et al., 2023). In contrast, RBV focuses on the strategic value of digital resources, such as technological infrastructure and human capital, which are key assets to maintain competitive advantage. In general, DCT provides the flexibility that organizations need to adapt. RBV explains how to use valuable digital resources strategically to improve organizational performance and ensure long-term competitiveness in the digital age.

On the other hand, digital transformation refers to the organizational process of integrating digital technology into enterprise operations, influencing internal and external interactions, and reshaping business models to improve performance and competitiveness (Tabrizi et al., 2019; Yao et al., 2024). Although digital leadership and digital transformation are closely related, they represent conceptually different structures and should not be treated interchangeably. Digital leadership refers to a set of leadership capabilities and behaviors that enable organizations to clearly express their digital vision, mobilize technology and human resources, and guide employees to adapt to digitalization (Ye, 2025). It mainly operates at the strategic and behavioral levels, shaping how organizations respond to digital challenges and opportunities. In contrast, digital transformation represents an organizational process and result, reflecting the degree to which digital technology is embedded in business operations, service delivery and value creation mechanisms (Matarazzo et al., 2021; Nazara et al., 2024; Soto Setzke et al., 2023). It is embodied through changes in workflow, organizational structure and business model. Therefore, digital leadership can be viewed as a driving force, while digital transformation captures the realization of organizational change.

Furthermore, the latest progress in digital leadership and digital transformation highlights its role in improving digital strategy (Yao et al., 2024), digital capabilities (Benitez et al., 2022), and resilience (AlNuaimi et al., 2022; Benitez et al., 2022). In addition, a large number of studies have highlighted the importance of leadership in fostering a digital culture and promoting the integration of IT resources (Alabdali et al., 2024; Benitez et al., 2022). Although more and more research focuses on the impact of leadership in advancing digital projects, empirical research examining the direct impact of digital leadership on digital transformation, especially in high-contact service sectors (such as hospitality) is still significantly insufficient. Preliminary studies have shown that executives who actively adopt digital transformation and foster a cooperative, technology-driven atmosphere are more likely to accelerate the success of the digital transformation initiative (AlNuaimi et al., 2022). Integrating DCT and RBV (Barney, 1991; Teece et al., 1997), this study argues that digital leadership supports transformation by continuously adjusting digital resources to adapt to changing market demands. Based on this, we put forward the following research hypothesis:

H1: Digital leadership positively affects digital transformation.

### 2.2 Digital leadership, job crafting, and digital transformation

Job crafting involves employees actively modifying the tasks, interpersonal dynamics, and cognitive meaning of their work to ensure that their roles are better aligned with their skills, values, and goals (Wrzesniewski and Dutton, 2001). These employees are more inclined to seek challenges, redefine task boundaries, and build resource networks required for performance and adaptability, thus showing greater flexibility and responsiveness in a complex and changing environment (Wong et al., 2021). Theoretically, job crafting is an important means for employees to respond to change and promote innovation (Petrou et al., 2012). According to DCT (Teece et al., 1997), job crafting can be understood as a dynamic capability at the micro level. Employees seize emerging opportunities by perceiving changes in the digital work environment and reconfigure their work roles accordingly. At the same time, from the perspective of the RBV (Barney, 1991), employees’ job crafting helps to develop valuable and difficult-to-imitate human resources and behavior resources, so as to enhance organizational adaptability and support sustainable competitive advantage in the digital environment. This enhances their ability and confidence to manage uncertainty and turns employees into important strategic assets that drive the digital transformation of the organization. This proactive behavior is usually rooted in an authorization atmosphere. Leaders create conditions for employees to reshape their work through trust, empowerment, exploration and innovation (Kim and Beehr, 2020). Through a clear digital vision, investment in employees’ technical capabilities, and legalization of experimentation, digital leadership shapes employees’ perception of autonomy and role flexibility, reduces the perceived risk of job role modification, and ultimately promotes job crafting in digital work situations (Ye, 2025). Specifically, empowered employees tend to optimize task processes, proactively seek the necessary resources, and redesign service processes to meet the requirements of digital transformation (Wang et al., 2025b). Although the role of leadership in promoting organizational change has been widely recognized, there is still a lack of empirical research on how digital leadership motivates employees to craft their job. In a service-oriented sector characterized by significant technological integration, clarifying this link can not only address the lack of research, but also provide practical insights for improving employee flexibility and the efficiency of digital transformation. Based on RBV (Barney, 1991), we believe that digital leadership can effectively activate employees’ job crafting by creating an environment that supports resource allocation and role refactoring.

Previous studies have shown that employee job crafting can improve adaptability, work engagement and innovation in many organizational contexts (Petrou et al., 2012; Tho, 2022). In particular, job crafting has proven to enhance firms’ digital resilience, improve service performance in the hospitality sector, and facilitate manufacturing’s transition to Industry 4.0 (Wang et al., 2025b; Yadav and Dhar, 2021; Zhu et al., 2022). However, the current literature fails to fully explore how employees can transform the change signals conveyed by digital leadership into specific job adjustment behaviors through job crafting to support the goal of digital transformation (Zhu et al., 2022). This research gap emphasizes the need to further study the proactive behavior of employees, such as redefining role boundaries and actively accessing resources, and how to enhance the adaptability of organizations in the dynamic digital environment, especially in service-oriented and customer-centric industries. A deep understanding of this relationship is essential for developing effective leadership strategies, motivating employees and promoting digital transformation from bottom to top. Accordingly, the following hypotheses are proposed on the digital leadership process, especially on how employees internalize the change signals conveyed by leaders and transform them into specific behaviors that promote organizational change.

H2a: Digital leadership positively affects employee job crafting.

H2b: Employee job crafting positively affects digital transformation.

H2c: Employee job crafting mediates the relationships between digital leadership and digital transformation.

### 2.3 Digital leadership, innovative work behavior, and digital transformation

Innovative work behavior refers to employees’ generation, promotion, and implementation of innovative ideas to improve work performance, which is a key mechanism for realizing digital transformation (Jong, 2007). Employees actively respond to complex challenges at work, propose novel digital solutions, advocate for the application of technology, and actively experiment with new tools (Zia et al., 2025). This is particularly important in service-oriented and technology-centric sectors because it places higher demands on agile response and continuous innovation. Existing research shows that digital leadership can not only motivate employees to identify innovation opportunities and emerging technologies through the skilled use of technology and data, but also motivate employees to actively test solutions and transform ideas into actionable work improvements, thereby enhancing the organizations’ innovation ability and adaptability (Ahmed et al., 2024; Mihardjo et al., 2019). In addition, digital leadership has the ability to navigate in a rapidly changing digital environment, identify and cultivate employees’ innovative potential, and guide teams to adopt advanced practices, thereby aligning individual creative contributions with organizational strategic goals and enhancing overall adaptability (Wang et al., 2022). Although leadership has been demonstrated to significantly impact employees’ innovative work behavior in technology-intensive sectors (Ahmed et al., 2024; Zia et al., 2025), research on how digital leadership specifically activates innovative behavior in service-oriented digital environments is still relatively scarce. According to RBV (Barney, 1991), the innovative work behavior of employees constitutes the micro-foundation of an organization’s capacity to perceive changes, adapt capabilities, and reorganize resources. Digital leadership, by creating an open and inclusive atmosphere, provides the critical conditions for employees to engage in problem-oriented exploratory behaviors and drive continuous innovation at the organizational level.

Furthermore, the present study reveals substantial deficiencies in comprehending the mediating variables connecting digital leadership and digital transformation. For instance, previous studies have identified various potential mediating variables, including digital strategy consensus (Yao et al., 2024), organizational resilience (AlNuaimi et al., 2022), trust, and innovative work performance (Weber et al., 2022). Nevertheless, empirical investigations regarding the mediation function of innovative work behavior between digital leadership and digital transformation remains limited. At the same time, the current research pays insufficient attention to employee-driven innovation behavior, and employee-driven innovation behavior is crucial to achieving leadership effectiveness in digital transformation, especially in the service industry (Busulwa et al., 2022). Therefore, based on the background of China’s service industry, this study empirically examines the mediating role of innovative work behavior in the process of digital transformation driven by digital leadership. Therefore, we propose the following hypothesis:

H3a: Digital leadership positively affects employee innovative work behavior.

H3b: Employee innovative work behavior positively affects digital transformation.

H3c: Employee innovative work behavior mediates the relationship between digital leadership and digital transformation.

### 2.4 Digital transformation and digital-enabled task performance

Digital transformation refers to the deep reconstruction of organizational processes, technical systems and business models through the strategic integration of digital technologies (AlNuaimi et al., 2022; Loebbecke and Picot, 2015). This process helps enhance the operational agility of enterprises, optimize work processes, and reshape task structures, thereby improving efficiency and responsiveness (Chan et al., 2019; Probojakti et al., 2025). Digital transformation usually emerges from the need to improve operational inefficiency and optimize task structures, enabling enterprises to more effectively mobilize and coordinate digital resources to enhance employees’ task performance (Qiao et al., 2024). Based on DCT (Teece et al., 1997), digital transformation gives organizations the ability to detect market volatility, leverage technological advances, and reorganize internal processes to deftly respond to change. The introduction of digital technologies such as automation, data decision-making and real-time communication tools into organizations can profoundly change the execution mode of organizational tasks, so as to improve individual performance (Chen, 2018; Correani et al., 2020).

Digital-enabled task performance emphasizes the ability of employees to improve work efficiency and task quality by using advanced digital technologies (such as big data analysis; Shao et al., 2024). This performance depends not only on whether the task is clear and whether the technical resources are in place, but also on the combined effect of data-driven culture and employee digital self-efficacy (Shao et al., 2024). It comprehensively reflects the quality of work, response speed, problem solving and adaptation level of employees in different complexity tasks. Studies have shown that digital transformation is significantly related to employee performance improvement (Qiao et al., 2024), in particular, through system integration and data visualization, task complexity and ambiguity can be reduced, and workflow operation can be accelerated. For instance, companies completing digital transformation often equip their front-line employees with automated tools to get things done faster and better in service-intensive scenarios. In addition, in a highly digitized operational environment, the organizational authorization mechanism strengthens employees’ digital skills and internal innovation capabilities (Blanka et al., 2022). Although the value of digital transformation to organizations has been recognized, empirical evidence on employees’ task performance is still insufficient, especially in service-oriented high-contact industries. Clarifying how transformation translates into measurable personal productivity gains remains to be explored. Based on the above theoretical perspectives and literature insights, combined with DCT (Teece et al., 1997), this study examines digital-enabled task performance, and propose subsequent hypotheses:

H4: Digital transformation positively affects employee digital-enabled task performance.

H5: Digital transformation mediates the relationship between digital leadership and employee digital-enabled task performance.

H6: Job crafting and digital transformation serially mediate the relationship between digital leadership and employee digital-enabled task performance.

**Figure 1:**
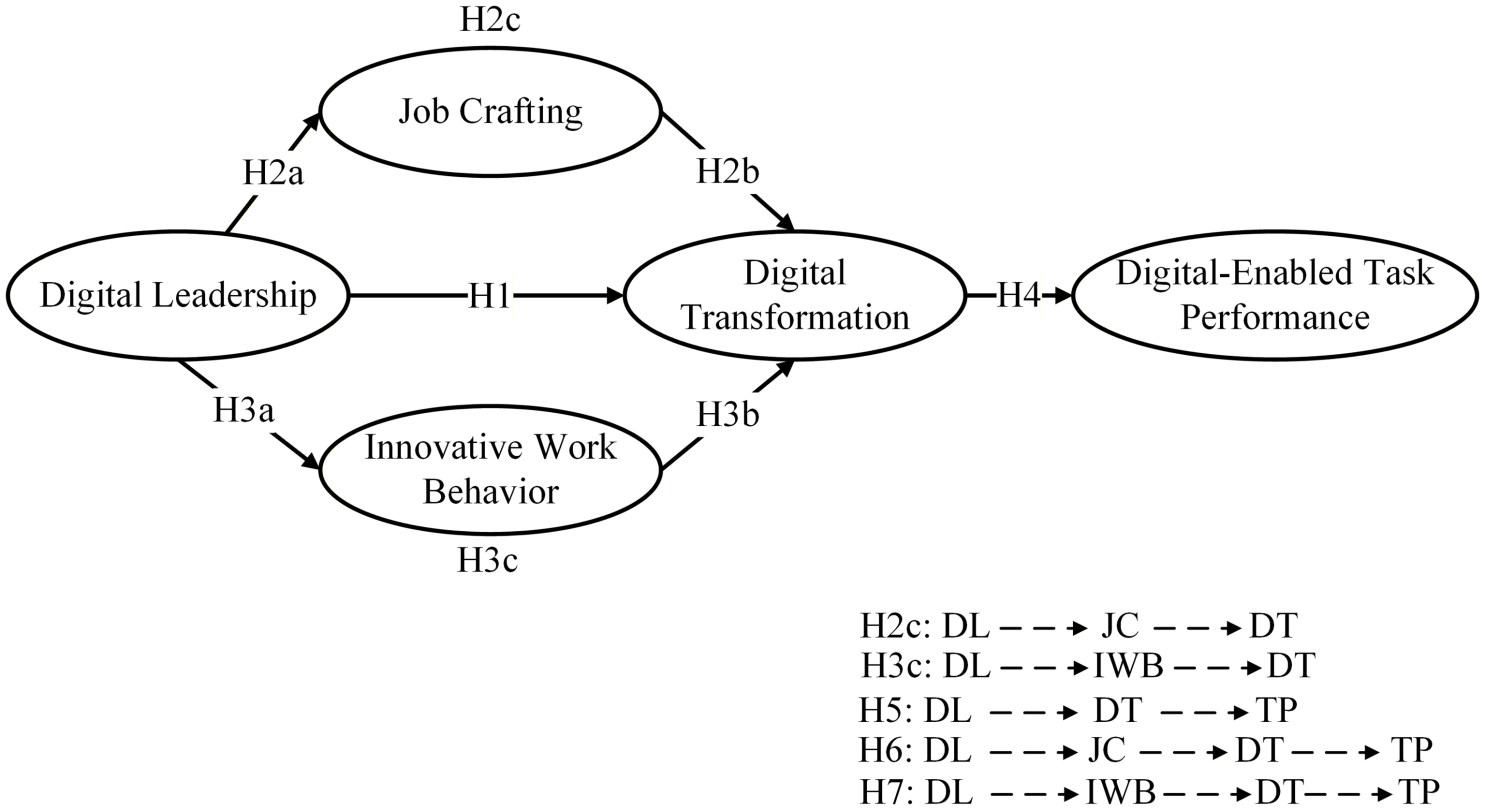
The theoretical framework of the research variables. Digital leadership (DL), job crafting (JC), innovative work behavior (IWB), digital transformation (DT), digital-enabled task performance (TP); solid arrows indicate direct paths, dashed arrows indicate indirect paths through mediating effects.

H7: Innovative work behavior and digital transformation serially mediate the relationship between digital leadership and employee digital-enabled task performance (Figure 1).

## 3 Methodology

### 3.1 Instrument

In this study, multiple validated scales were used to measure the core variables, and appropriate adjustments and adaptations were made in combination with the research situation.

Digital leadership was measured using six items developed by Zeike et al. (2019). The scale covers individuals’ attitudes, abilities and leadership behaviors towards the use of digital tools. Among them, the first three focus on the tendency to use digital tools, and the last three assess the ability to guide and coordinate in digital transformation. An example is: “My leader believes that the use of digital tools is enjoyable and valuable”.

Job crafting was measured with nine items developed by Niessen et al. (2016) to measure employees’ proactive behavior in adjusting work tasks, interpersonal relationships, and cognitive meaning. An example is: “I will focus on completing specific tasks”.

Innovative work behavior adopts nine items developed by Janssen (2000), which correspond to the three stages of creative generation, promotion and implementation, and are widely used in empirical research on employee innovative behavior. Examples include: “I often put forward new ideas on current hot topics” (idea generation), “I actively seek support from colleagues or superiors for innovative concepts” (idea promotion), “I can effectively transform innovative ideas into practical applications” (idea realization).

Digital transformation uses five items proposed by AlNuaimi et al. (2022), Chen and Wang (2024), and Odai et al. (2026) to evaluate employees’ subjective perception of the degree of digital transformation within the organization, covering dimensions such as data collection, process optimization, and information sharing. One example is: “My organization is committed to digitizing all tasks to the greatest extent possible”.

Digital-enabled task performance, supported by digitalization, is measured using three scales proposed by Zhang (2017), focusing on employees’ ability to enhance work efficiency and quality through intelligent technologies or big data analysis. For example: “Big data analysis has significantly improved the quality of my work.”

This study incorporates control variables to consider possible confounding factors at the organizational and individual tiers, including firm size (number of employees), years of establishment, and the age, gender, education level and years of work of employees (Shao et al., 2024). All assessments were conducted using the 5-point Likert scale, scoring from 1 (strongly disagreed) to 5 (strongly agreed). According to the suggestion of Memon et al. (2023), five frontline hospitality employees were recruited to pre-test the questionnaire before the official release to ensure the clarity of the language and the contextual relevance of the items. In order to ensure the linguistic and conceptual equivalence between the original English version and the Chinese version of the scale, we employed a forward-backward translation method. The scale was first translated into Chinese by two bilingual researchers with hotel management expertise, and then back translated into English by a third bilingual expert. Any discrepancies were resolved, the research team will resolve them through consultation. In addition, two management experts with associate professor titles conducted a content validity review of the adjusted scale, and no further revision was recommended during the review process.

### 3.2 Sampling and data gathering

This study adopts a purposeful snowball sampling strategy, which is especially suitable for visiting front-line employees working in digitally advanced hospitality organizations (Wiśniowski et al., 2020). Given the uneven distribution of digital transformation in hospitality industry across companies, employees with direct experience in the process of digital services constitute a relatively professional and difficult to reach group. Therefore, snowball sampling helps to identify respondents who meet specific research criteria and have relevant experience in digital transformation practice.

The target group includes full-time frontline employees with at least 1 year’s work experience in organizations with a high degree of digitalization. These organizations usually deploy technologies such as intelligent check-in and check-out systems, AI driven customer service, self-service kiosks and integrated digital management platforms. Frontline employees are particularly suitable for this study because they are directly involved in the implementation and daily use of digital systems, so they can have an in-depth understanding of employee level behavior mechanisms, including job crafting and innovative work behavior.

Data were collected through multiple channels to enhance sample diversity. Specifically, questionnaires are distributed through professional and industry-related networks on the WeChat platform and internal organizational channels (including human resources departments and internal communication systems). Participation is voluntary, anonymous and confidential, and provides a small incentive to improve response rates and data quality.

Although snowball sampling is appropriate in this context, it is not unlimited. Due to network-based recruitment, this method may be subject to selection bias, which could affect the representation of the sample. To alleviate this concern, participants are recruited through multiple independent entry points to reduce network homogeneity. In addition, the sample includes employees from organizations of different sizes, ages and operating sizes, which enhances the heterogeneity and improves the robustness of the research results. In addition, the focus on frontline employees ensures that respondents have relevant and experience based-knowledge of digital transformation practices.

In addition, some procedural remedies have been implemented to ensure data quality. The respondents clearly understood the purpose of the study and questionnaire items were randomly ordered to reduce the common method bias associated with item order. A total of 415 valid questionnaires were collected for follow-up analysis. These steps jointly ensure the situational applicability of the sample and the reliability of the data collected in the empirical test (Table 1).

**Table 1:** Basic characteristics (*N* = 415).

Category	Subcategory	Frequency	%
Individuals’ data			
Gender	Male	230	55.40
	Female	185	44.60
Age (years)	20–30	29	7.00
	31–40	216	52.00
	41–50	122	29.40
	More than 51	48	11.60
Education	Up to high school or equivalent	144	34.70
	Bachelors	150	36.10
	Postgraduate and above	121	29.20
Experience (years)	Less than 5	91	21.90
	6–10	158	38.10
	11–15	32	7.70
	Up to 15	134	32.30
Firms’ data			
No. of employees	Under 200	92	22.20
	201–500	120	28.90
	501–1,000	75	18.10
	Above 1,000	128	30.80
Age (years)	Less than 5	77	18.60
	5–9	122	29.40
	10–19	57	13.70
	20–29	77	18.60
	More than 30	82	19.80

### 3.3 Common method bias

Common method bias (CMB) may come up from inflated or distorted associations of the relationship between variables when collecting data from the same source (Jordan and Troth, 2020). This problem is particularly common in the study of self-report questionnaires (Memon et al., 2023). This study follows the recommendations of Jordan and Troth (2020) and Podsakoff et al. (2003) to implement procedural and statistical remedies to minimize the impact of potential CMB. In this study, a number of preventive measures were included in the design and management of the questionnaire. First, we recruited 10 front-line employees to participate in the pre-test of the questionnaire in a highly digital working environment to ensure that the questionnaire items were clear and contextually appropriate. Second, all participants had a clear understanding of the study objectives before completing the questionnaire, and ensured anonymity and confidentiality to reduce social desirability bias. Thirdly, the questionnaire design used random ordering and mixed presentation of items with different dimensions, which avoids the systematic deviation caused by the effect of item order. In addition, in terms of statistical tests, this study further used Harman (1976) single-factor test to evaluate CMB. The study found that no factor explained more than 40.72% of the total variance and did not reach the 50% threshold, indicating that the data in this study did not show significant CMB concerns. However, it is worth noting that Harman’s test has been criticized for its limited sensitivity in detecting method bias. Therefore, the interpretation of the results should be combined with the procedural remedies taken. In conclusion, the combination of process control and statistical evaluation provides reasonable confidence that common method bias is unlikely to substantially affect the effectiveness of the research results.

## 4 Analysis of data and findings

In this study, Smart PLS 4.0 software was used to conduct partial least squares structural equation modeling for hypothesis testing. PLS-SEM is appropriate for this study for three reasons. Firstly, PLS is widely recommended for handling highly predictive, complex structures, and multiple mediating variables (Richter et al., 2015; Roldán and Sánchez-Franco, 2012). Given that the models in this research include multiple variables and parallel continuous mediation paths, PLS is considered to be the most appropriate analytical technique. Secondly, the main research objectives are predictive and explanatory, focusing on how digital leadership affects digital transformation and employee performance through behavioral mechanisms. Thirdly, PLS-SEM is very suitable for processing non-normal data distribution and is robust for medium-sized samples, such as the 415 observations used in this study. Following the two-stage approach recommended by Anderson and Gerbing (1988), we first evaluate the measurement model to test the reliability and validity of the latent construct before testing the structural relationship. The evaluation of the measurement model mainly focuses on internal consistency reliability and validity. In the subsequent stage, the structural model is tested, focusing on the path relationship between variables and the significance of the mediating effect.

### 4.1 Measurement model and multicollinearity

All constructs show strong internal consistency. The alpha (*α*) and CR values of all unobserved variables were higher than the recommended criterion of 0.70 (Hair et al., 2017), indicating satisfactory reliability. The convergent validity was also established. All standardized factor loadings were above 0.70, and the average variance extraction (AVE) value exceeded the recommended minimum value of 0.50 (Fornell and Larcker, 1981), implying that the variance of the items was fully explained by each construct.

Furthermore, discriminant validity is established by 3 criteria: (1) inter-construct correlations were below 0.85 (Kline, 1998); (2) the square root of its AVE for each construct is greater than its correlation with all other constructs (Fornell and Larcker, 1981); (3) The heterotrait-monotrait ratio (HTMT) between all structures is lower than 0.85 (Henseler et al., 2015; Voorhees et al., 2016), which further confirmed the uniqueness of each structure. Similarly, the variance inflation factor (VIF) is calculated to evaluate potential multicollinearity problems. The results show that all VIF values range from 2.10 to 6.96, which is far lower than the conservative standard 10 (Hair et al., 2019), indicating that there is no significant risk of multicollinearity in this research model.

**Figure 2:**
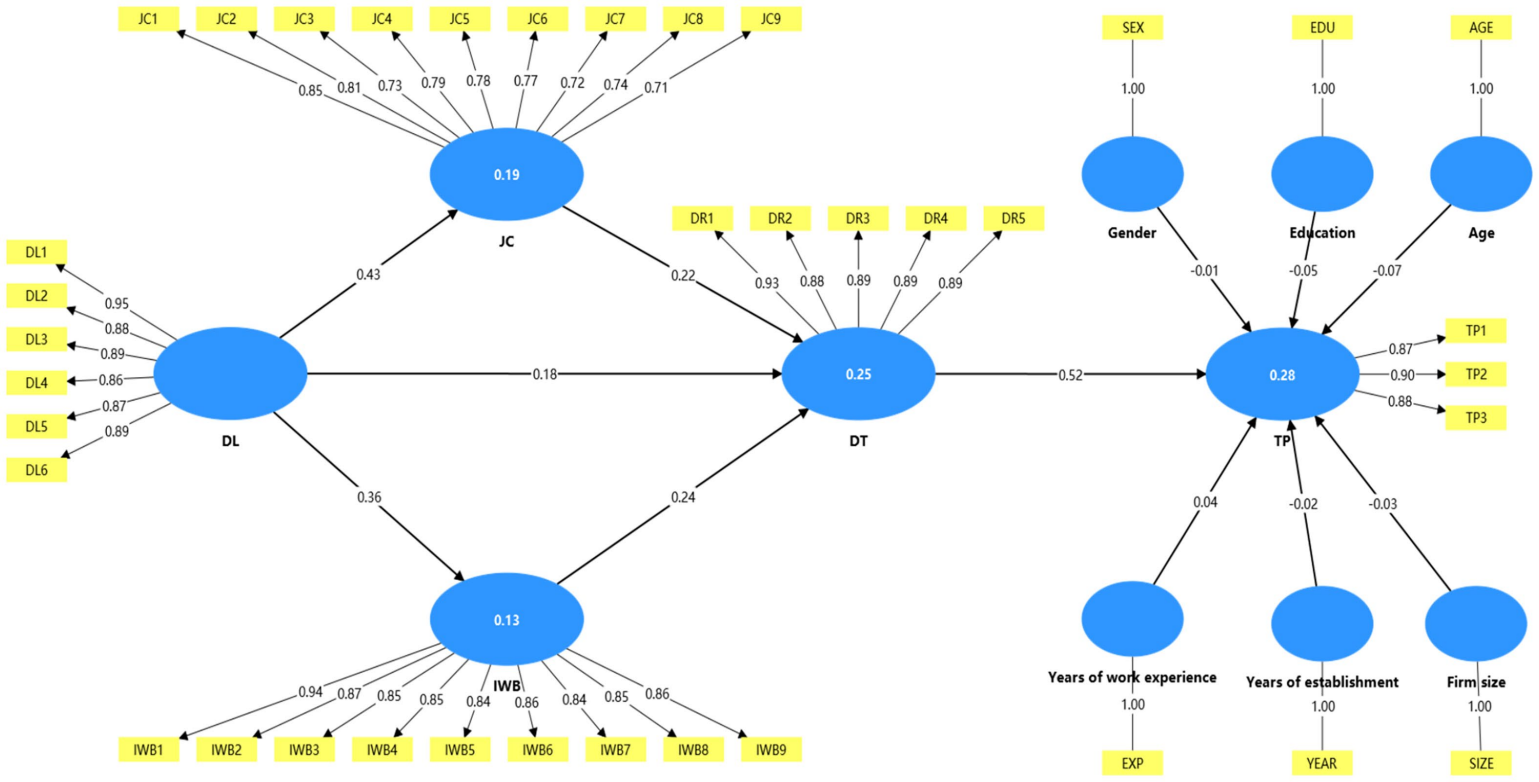
Structural model. The value on the arrow represents the normalized path coefficient, and the value in the circle represents *R*^2^.

In order to improve transparency, the measurement model is shown in Figure 2, which shows the relationship between the potential structure and its corresponding indicators, as well as the standardization factor load (Table 2).

**Table 2:** Model evaluation.

Constructs	Loadings	VIF	*α*	CR	AVE
DL	0.86–0.95	2.74–6.40	0.95	0.95	0.79
DT	0.88–0.93	2.97–4.58	0.94	0.94	0.80
IWB	0.84–0.94	2.81–6.96	0.96	0.96	0.75
JC	0.71–0.85	2.62–6.40	0.91	0.92	0.59
TP	0.87–0.90	2.10–2.49	0.86	0.86	0.79

Fornell–Larcker	1	2	3	4	5
DL	**0.89**				
DT	0.36	**0.90**			
IWB	0.36	0.41	**0.86**		
JC	0.43	0.42	0.52	**0.77**	
TP	0.44	0.52	0.47	0.51	**0.89**

HTMT	1	2	3	4	5
DL					
DT	0.38				
IWB	0.37	0.43			
JC	0.46	0.45	0.56		
TP	0.49	0.57	0.51	0.58	

Diagonal bold values = √AVE.

### 4.2 Structural model

After establishing the appropriateness of the measurement model, we evaluated the structural model to test the relationship and mediating effect of the hypothesis. The structural model evaluation mainly focuses on the path coefficient and its statistical significance, as well as the explanatory power of the model. Table 3 demonstrates that DL has a positive and statistically significant association with DT (*β* = 0.18, *t* = 2.90, *p* < 0.05), confirming H1; DL is also positively related to JC (*β* = 0.43, *t* = 9.10, *p* < 0.05) and IWB (*β* = 0.36, *t* = 6.70, *p* < 0.05), supporting H2a and H3a. In addition, both JC (*β* = 0.22, *t* = 3.44, *p* < 0.05) and IWB (*β* = 0.24, *t* = 3.89, *p* < 0.05) are positively associated with DT, respectively supporting H2b and H3b. DT, in turn, shows a positive relationship with TP (*β* = 0.52, *t* = 10.77, *p* < 0.05), supporting H4. Therefore, all direct path hypotheses have received empirical support.

To test the proposed mediating effects (H2c, H3c, H5, H6, H7), indirect effects were further assessed. The study indicates that, despite the inclusion of mediating variables in the model, the direct effect of DL on DT remains significant (*β* = 0.18, *t* = 2.90, *p* < 0.05), indicating partial mediation. Furthermore, DL shows positive indirect effects on DT via JC (*β* = 0.09, *t* = 3.20, *p* < 0.05) and IWB (*β* = 0.09, *t* = 3.26, *p* < 0.05), supporting H2c and H3c. Similarly, DT mediates the association from DL to TP (*β* = 0.09, *t* = 2.77, *p* < 0.05), supporting H5. In addition, the sequential mediation effects through JC and DT (*β* = 0.05, *t* = 2.89, *p* < 0.05), and through IWB and DT (*β* = 0.04, *t* = 2.96, *p* < 0.05), are also statistically significant, thereby supporting H6 and H7.

**Table 3:** Hypothesis results.

Hypothesis	Paths	*β*	SD	*t* value	*p* value	2.5% CI	97.5% CI
H1	DL -> DT	0.18	0.06	2.90	0.00	0.06	0.29
H2a	DL -> JC	0.43	0.05	9.10	0.00	0.33	0.52
H2b	JC -> DT	0.22	0.06	3.44	0.00	0.09	0.33
H2c	DL -> JC -> DT	0.09	0.03	3.20	0.00	0.04	0.15
H3a	DL -> IWB	0.36	0.05	6.70	0.00	0.25	0.46
H3b	IWB -> DT	0.24	0.06	3.89	0.00	0.12	0.35
H3c	DL -> IWB -> DT	0.09	0.03	3.26	0.00	0.04	0.14
H4	DT -> TP	0.52	0.05	10.77	0.00	0.41	0.60
H5	DL -> DT -> TP	0.09	0.03	2.77	0.01	0.03	0.16
H6	DL -> JC -> DT -> TP	0.05	0.02	2.89	0.00	0.02	0.08
H7	DL -> IWB -> DT -> TP	0.04	0.01	2.96	0.00	0.02	0.08

^* *p* < 0.05, ** *p* < 0.01, *** *p* < 0.001.^

It is worth noting that the magnitude of some indirect effects is relatively small (e.g., *β* = 0.05). However, such effect sizes are not uncommon in behavior research involving complex mediation processes. In this case, even modest impact can provide meaningful insight into the potential mechanisms that link digital leadership to digital transformation and performance outcomes. These findings show that employee-driven behaviors, such as job crafting and innovative work behavior, play a subtle but important role in transformation leadership influence into organizational change, rather than indicating weak relationships. Overall, the research results emphasize that these mechanisms, although modest in size, contribute to explain how digital leadership is associated with digital transformation and digital-enabled task performance (see Table 3).

## 5 Discussion and contributions

The results show the key function of digital leadership in stimulating innovative work behavior and job crafting, which are two complementary active behaviors to promote digital transformation and improve task performance. This view helps deepen the understanding of leadership in the digital age, and emphasizes how digital leadership can actively shape employee behavior, so as to cultivate innovation culture and promote organizational transformation.

The results further indicate how digital leadership can influence employees’ perceptions of autonomy and role flexibility by providing clear vision and encouraging experiments. These concepts in turn trigger proactive job crafting, which is essential to adapt to the digital transformation. This finding emphasizes the importance of employee-driven action in the process of digital transformation, which is consistent with the view that leadership is the key driver of organizational change, not only at the strategic level, but also through the micro level behavior of employees’ daily implementation.

When these findings are compared with previous literature, this study provides a unique perspective on the role of digital leadership in employee behavior. Previous studies emphasized the importance of leadership in promoting digital transformation, but focused on leadership from an organizational perspective (Leso et al., 2023). In contrast, this study emphasizes the micro mechanism of the impact of digital leadership on individual behavior, thus extending the applicability of leadership theory to the digital environment. In addition, our research integrates DCT and RBV (Barney, 1991; Teece et al., 1997), emphasizing how job crafting can be conceptualized as a dynamic capability, so that employees can perceive, master and reconfigure digital resources.

In addition, different from the previous view that innovative work behavior is regarded as a passive response to performance pressure (Arun Kumar and Lavanya, 2024), our research results show that this is an active mechanism through which leadership exerts influence. Specifically, digital leadership practices create an environment that empowers employees and encourages experimentation, encouraging them to participate in innovative activities. Digital leaders help employees develop innovative solutions by reducing perceived risks and encouraging errors, so as to enhance organizational adaptability and promote the process of digital transformation. Although it is emphasized that employee autonomy and independent innovation are the key factors for organizational success, few studies have explored how leadership practices can enhance innovative work behavior, especially in the context of digital transformation.

Finally, previous literature has not fully studied the role of innovative work behavior in digital-enabled task performance. Our results show that training employees’ innovative work behavior and job crafting can significantly improve task performance. By doing so, employees can replan processes, optimize workflow, and solve problems creatively in the digital environment. This supports the view that innovative work behavior is essential to adapt to technological progress, especially in industries such as hotels, where digital technology is the core of operations.

### 5.1 Theoretical contributions

This study has four theoretical contributions. This study integrates organizational and employee-level factors into a research framework, and addresses a fundamental question that is rarely studied in organizational effectiveness, that is, how organizations can successfully digitally transform and improve employees’ adaptive performance. This model reveals the path mechanism by which hospitality enterprises stimulate employees’ initiative and innovative behavior through digital leadership, thereby driving digital transformation and achieving high-level task performance.

Secondly, this study focuses on the employee-driven mechanism and clarifies the key behavioral foundations needed to achieve the transformation from digital leadership to performance improvement. In the context of the increasing emphasis on innovation and customer orientation in the hospitality industry, human capital has always been the core force driving digital transformation. However, current research rarely integrates job crafting and innovative work behavior into a unified analytical framework to fully elucidate the impact of employee behavior on the relationship between leadership and digital transformation (Ahmed et al., 2024; Wang et al., 2025b; Zia et al., 2025). This study combines two key employee behaviors and develops a behavioral path model to clarify how digital leadership motivates front-line employees to actively modify tasks and propose innovative solutions to achieve the dual goals of promoting transformation and improving performance. This study enhances the understanding of the mechanism of employee behavior in the field of leadership and organizational behavior, and also addresses a gap in prior research that ignore the effect of employee-level behavior on the nature of performance development (Chatterjee et al., 2023; Qiao et al., 2024).

Thirdly, the findings show that digital leadership is an important antecedent of employees’ job crafting and innovative work behavior, which reflects the adaptability and creativity of employees in the process of digital transformation. More importantly, the findings reinforce the conceptual distinction between digital leadership and digital transformation. Digital leadership is not equal to the result of transformation, but as a kind of empowerment, shaping employee-driven behavior mechanism, through which transformation can be carried out. In contrast, digital transformation captures the cumulative organizational changes caused by this impact. By clarifying this difference, this study provides a more accurate theoretical positioning of the relationship between leadership and transformation, while avoiding the common conceptual confusion in previous studies, thus promoting the development of digital leadership literature (AlNuaimi et al., 2022; Odai et al., 2026).

Finally, this study focuses on task performance, which is a key factor in the evolution of organizational performance priority, and is also an area of insufficient research in the literature. At present, there are few studies on how digital leadership affects the behavior of employees in the frontline service roles (Busulwa et al., 2022). These behaviors will affect the digital transformation of the organization and the performance results at the employee level. In addition, previous studies on the determinants of employee task performance mainly focused on the structural or organizational level, often ignoring the impact of employee-driven behavioral mechanisms on individual performance development (He et al., 2019). Especially in the service industry, employee behavior is closely related to performance. This study reveals that digital leadership can create an organizational atmosphere, enhance employees’ initiative and improve employees’ job performance. This discovery broadens the employee centered research perspective, highlights the substantial impact of employees’ proactive behavior on performance improvement strategies in the digital environment, and provides empirical support for employees’ role adaptation and value creation in the rapidly changing technology environment.

### 5.2 Managerial contributions

This research delivers operational implications for hotel decision-makers to help them simultaneously improve employee performance in the digital transformation process. First, service-oriented organizations must adjust their internal structure as soon as possible in order to transform digital transformation into higher employee performance (Chatterjee et al., 2023). The key is to activate the initiative of employees, especially job crafting and innovative work behavior. To this end, management needs to create a cultural atmosphere that encourages digital leadership, and support flexible and performance-centered human resources policies to align employee behavior with the organization’s digital goals. The empirical results show that digital leadership significantly encourages employees to engage in job crafting and innovative work behavior, thus achieving the dual improvement of organizational transformation and performance. For hotel companies in transition, relying on digital leadership can effectively guide employees’ creative input and adaptive performance, encourage employees to redesign tasks, test new technologies, clarify visions for change, and delegate decision-making power, so that employees can easily cope with operational and technical challenges in a rapidly changing digital environment.

The findings also suggest that if the hotel wants to achieve a successful transformation in the digital wave, it must incorporate digitization into the corporate culture and allow employees to take the initiative. With this ability, employees can effectively use digital tools, integrate them into service processes, and quickly adjust their work strategies under the impact of transformation. To this end, service enterprises should systematically plan and implement a set of capacity development programs for digital scenarios, which can not only provide employees with comprehensive tool training, but also strengthen employees’ ability to solve problems on the spot through situational exercises, and improve employees’ flexibility to adjust in the face of emergencies through cognitive training. When employees have behavioral agility, they can reorganize tasks and propose new ideas at any time to enhance the resilience of enterprises in digital service scenarios. At the same time, cross-functional collaboration platforms are also important for sustaining such innovative behavior. Managers should give priority to the deployment of digital collaborative systems, so that knowledge sharing and real-time feedback become the norm, so that employees’ job crafting and innovative work behavior are consistent with organizational strategy, and ensure that organizational transformation and employee performance are improved simultaneously. In addition, the digital system should be matched with the workflow. The hotel needs to regularly review the fit between employee behavior and the technical environment, identify friction points in a timely and optimize the integration strategy, so as to continuously empower employees and support the long-term advancement of digital transformation.

Hotel companies need to realize that the effectiveness of digital transformation will directly affect employee performance. Managers should prioritize digital projects with employee behavior as the core. By encouraging employees to actively engage in job crafting and innovative practices, digital tools can be truly embedded in service processes, thus driving the simultaneous improvement of operational efficiency and customer satisfaction (Chan et al., 2025; Chatterjee et al., 2023; Deng et al., 2022). In daily management, hotels can continuously monitor service operation, task completion and staff adaptation speed with advanced analysis tools, so as to timely capture changes in the digital environment and adjust strategies. An intelligent check-in, mobile concierge, and artificial intelligence-driven service tracking platform simplifies front-line processes, reduces redundant steps, and makes the front desk respond faster. At the same time, real-time communication and cross-departmental collaboration within the organization are strengthened, and these initiatives allow employees to gain more autonomy and clear boundaries of responsibility. In addition, digital management should be implemented within the organization, and an automated system should be established to record employee performance and their digital participation. This helps to more accurately evaluate employee performance and strengthen the linkage between leadership and employee effectiveness, thus laying the foundation for building an agile and efficient technology-driven service organization.

### 5.3 Limitations and future directions

This study has several limitations that should be acknowledged. First, the data are based on cross-sectional and self-report designs, which may limit the ability to establish causal inferences and introduce potential common method bias, despite the use of procedural and statistical remedies. Future research is encouraged to adopt multi-source or time-lagged design to strengthen causal inference and reduce potential method-related problems.

Second, the sample is from China’s hospitality industry, which may limit the external validity of the research results. The institutional environment, technological infrastructure and cultural background vary across countries and industries, which may affect the way digital leadership affects employee behavior and organizational transformation. Future research can expand this research by using cross-industry and cross-national samples, so as to enhance the generalizability and background robustness of the research results.

Third, this study relies on linear modeling method (PLS-SEM) to test the relationship between variables. Although it is suitable for testing complex mediation models, this method may not fully capture the potential non-linear, asymmetric or multi-level dynamics. Future research may consider alternative methods, such as Fuzzy-Set Qualitative Comparative Analysis (FSQCA) or Hierarchical Linear Model (HLM), to explore boundary conditions and more complex interaction patterns.

## Data Availability

The original contributions presented in the study are included in the article/Supplementary material, further inquiries can be directed to the corresponding author.
